# Crystal structure of benzene-1,3,5-tri­carb­oxy­lic acid–4-pyridone (1/3)

**DOI:** 10.1107/S2056989015017867

**Published:** 2015-10-03

**Authors:** Selena L. Staun, Allen G. Oliver

**Affiliations:** a Department of Chemistry and Biochemistry, University of Notre Dame, Notre Dame, IN 46556-5670, USA

**Keywords:** crystal structure, hydrogen-bond framework, polymorph

## Abstract

Slow co-crystallization of a solution of benzene-1,3,5-tri­carb­oxy­lic acid with a large excess of 4-hy­droxy­pyridine produces an inter­penetrating, three-dimensional, hydrogen-bonded framework consisting of three 4-pyridone and one benzene-1,3,5-tri­carb­oxy­lic acid mol­ecules, C_9_H_6_O_6_·3C_5_H_5_NO. This structure represents an ortho­rhom­bic polymorph of the previously reported *C*-centered, monoclinic structure [Campos-Gaxiola *et al.* (2014 ▸). *Acta Cryst.* E**70**, o453–o454].

## Abstract 2

A 5:1 mixture of 4-hy­droxy­pyridine with benzene 1,3,5-tri­carb­oxy­lic acid in methanol yields the title hydrogen-bonded framework compound. This compound crystallizes in the ortho­rhom­bic space group *P*na2_1_ and is a polymorph of the same stoichiometric species, reported in *C*c.

## Chemical context   

We have been inter­ested in the co-crystallization properties of benzene carb­oxy­lic acid derivatives (namely: benzene-1,4-di­carb­oxy­lic acid and benzene-1,3,5-tri­carb­oxy­lic acid) with 3- and 4-hy­droxy­pyridines (Staun & Oliver, 2012 ▸, 2015 ▸; Bhogala *et al.*, 2005 ▸). A variety of 3-hy­droxy­pyridine co-crystallants with benzene carb­oxy­lic acids have already been reported and we discontinued pursuit of those materials (Shattock *et al.*, 2008 ▸). Both 4-hy­droxy­pyridine and benzene-1,3,5-tri­carb­ox­ylic acid have been used extensively in both metal-organic frameworks as well as suitable donor/acceptor species in crystal engineering (see for example: Castillo *et al.*, 2001 ▸; Qian *et al.*, 2014 ▸). Recently we reported the characterization of the 1:1 co-crystallant 4-hy­droxy­pyridinium 3,5-di­carb­oxy­benzoate (Staun & Oliver, 2015 ▸). We also discovered that from similar preparative conditions (slow evaporation from methanol) with a larger molar ratio of 4-hy­droxy­pyridine to benzene-1,3,5-tri­carb­oxy­lic acid (BTC) a new species could be obtained; reported herein. A comparison of the structure with the Cambridge Structure Database revealed an identical structural motif, albeit in a different crystal system (Campos-Gaxiola *et al.*, 2014 ▸). Thus, we report the ortho­rhom­bic polymorph of benzene-1,3,5-tri­carb­oxy­lic acid–4-pyridone (1/3).
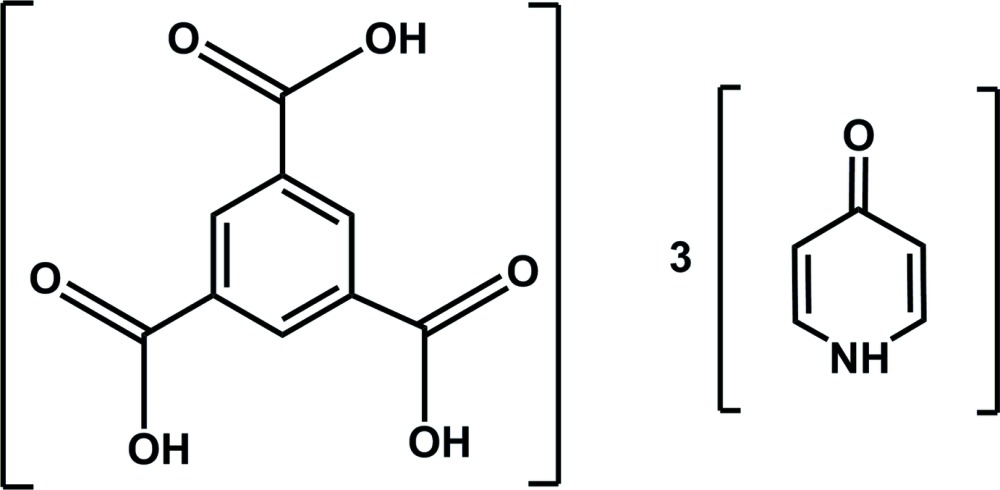


## Structural commentary   

The dihedral angles formed by the carb­oxy­lic acid moieties with respect to the benzene ring are 2.95 (16), 6.23 (10) and 10.28 (18)°. These are comparable with those for the previously reported polymorph of this compound [3.9 (2), 9.3 (2), and 13.3 (2)°; Campos-Gaxiola *et al.*, 2014 ▸]. It should be noted that the 4-hy­droxy­pyridine has undergone rearrangement from a hy­droxy­pyridine to the pyridone form of the mol­ecule as previously observed (Tyl *et al.*, 2008 ▸). The 4-pyridone C—O bond distances range from 1.280 (8) to 1.295 (8) Å. These distances are comparable with previously reported examples of this mol­ecule (Staun & Oliver, 2012 ▸; Tyl *et al.*, 2008 ▸). Inspection of the bond distances about each pyridone ring shows a slight tendency for the C—C bonds α to the nitro­gen [1.347 (12) to 1.371 (11) Å] to be shorter than those to the carbonyl carbon [1.410 (11) to 1.421 (10) Å]. This supports the proposed formal, localized double bond along the ‘edges’ of the pyridone ring.

Two of the three 4-pyridone rings are co-planar with the benzene tri­carb­oxy­lic acid moiety, similar to that of the previously reported structure (Campos-Gaxiola *et al.*, 2014 ▸). The remaining 4-pyridone is essentially perpendicular to this plane, also similar to the Campos-Gaxiola structure (Table 1 ▸).

## Supra­molecular features   

**Figure 1:**
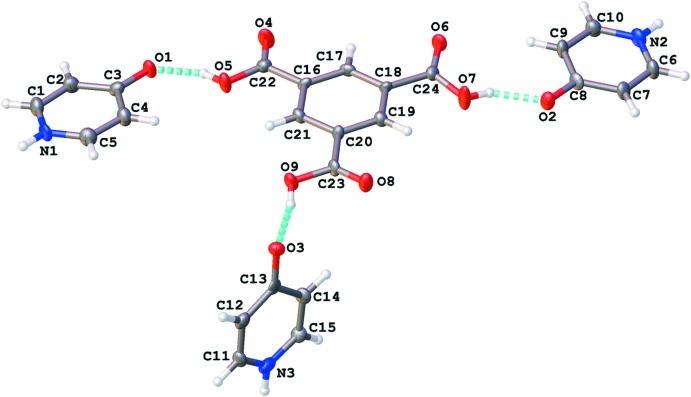
Labeling scheme for title compound. Atomic displacement ellipsoids are depicted at the 50% probability level. Dashed lines represent hydrogen bonds within the asymmetric unit.

**Figure 2:**
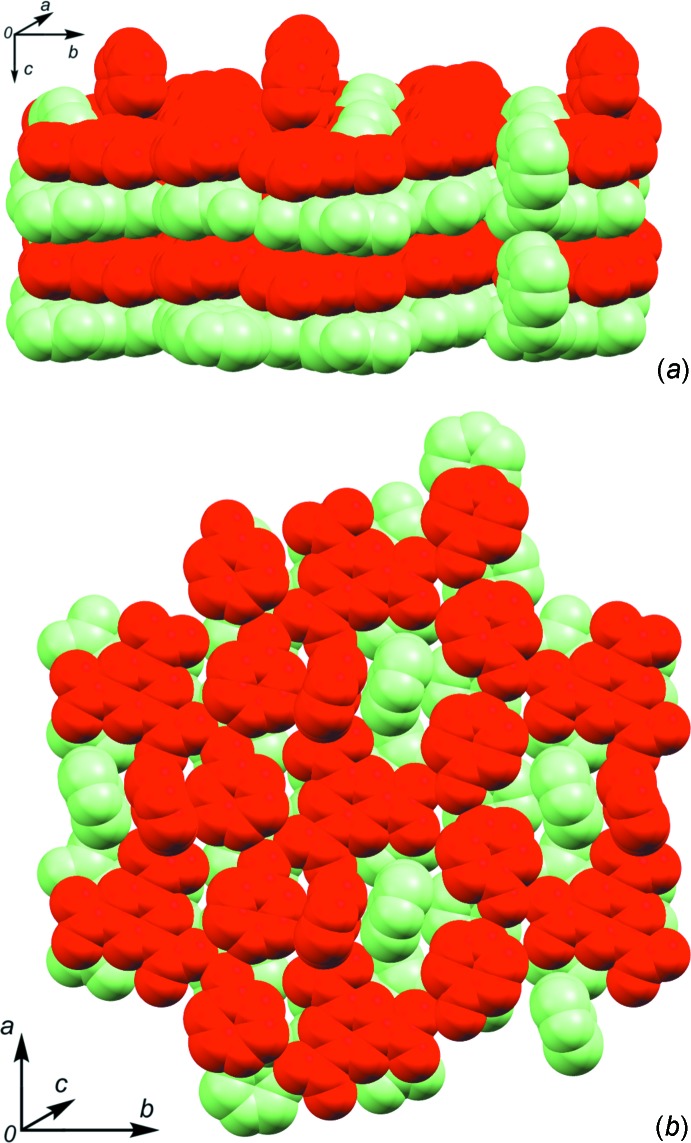
Space-filling views displaying the inter­penetrating networks (*a*) along the *a* axis; (*b*) along the *c* axis.

Each of the pyridone mol­ecules forms a hydrogen-bonded chain of symmetry-related mol­ecules. N1 and N2 form hydrogen bonds to O1^i^ and O2^ii^, respectively, related by the crystallographic *n*-glide [symmetry codes: (i) *x* − 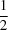, −*y* + 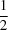, *z*; (ii) *x* + 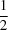, −*y* + 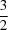, *z*]. N3 forms hydrogen bonds to O3^iii^ and O6^iv^ related by translation along the crystallographic *c*-axis and the [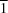
01] direction, respectively [symmetry codes: (iii) *x*, *y*, *z* + 1; (iv) *x* − 1, *y*, *z* + 1). Thus N3 forms a bifurcated hydrogen bond. These chains of hydrogen-bonded pyridone mol­ecules are bridged by the BTC mol­ecule. Each carb­oxy­lic acid moiety on BTC donates a hydrogen bond to a nearby pyridone carbonyl oxygen (Fig. 1 ▸, Table 2 ▸). These O_COOH_⋯O_py_ contacts are short for O—H⋯O contacts indicating strong inter­molecular hydrogen bonding. As a result of the N3 pyridone being oriented almost perpendicular to the plane of the other three mol­ecules, the resulting architecture is a three-dimensional hydrogen-bonded network. The BTC, N1 and N2 pyridone mol­ecules form a graph-set 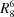
(44) ring that is parallel with the *ab* plane (Macrae *et al.*, 2008 ▸). This corresponds with that observed by Campos-Gaxiola *et al.* The BTC and N3 pyridone form an 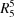
(30) ring that is perpendicular to the previous ring. Further inspection of this network reveals that there are two independent, inter­penetrating networks (Fig. 2 ▸). The BTC mol­ecules in the two networks form typical slipped π–π-stacks [*C_g_*⋯*C_g_* = 3.592 (5) Å, *C_g_*⋯*perp* = 3.302 (4) Å; *C_g_* represents the center of gravity of the ring, *perp* is the shortest perpendicular distance; Spek, 2009 ▸]. Other potential π–π contacts are beyond 4 Å. Due to the efficient packing of these mol­ecules there is a significant number of close C—H⋯O contacts, primarily between pyridone carbon atoms and carb­oxy­lic acid oxygen atoms, with one notable example being a contact from C9 to O3^v^ [symmetry code: (v) *x* + 1, *y*, *z*].

## Database survey   

A search in the Cambridge Structural Database (CSD, Version 5.36 plus 3 updates; Groom & Allen, 2014 ▸) for 4-hy­droxy­pyridine with benzene-1,3,5-tri­carb­oxy­lic acid produced only one hit. The compound is closely related to the title compound, namely: benzene-1,3,5-tri­carb­oxy­lic acid-pyridin­ium-2-olate (1/3) (Campos-Gaxiola *et al.*, 2014 ▸). However, the structure is reported to be in the monoclinic space group *C*c.

## Comparison with the structure of the monoclinic polymorph   

**Figure 3:**
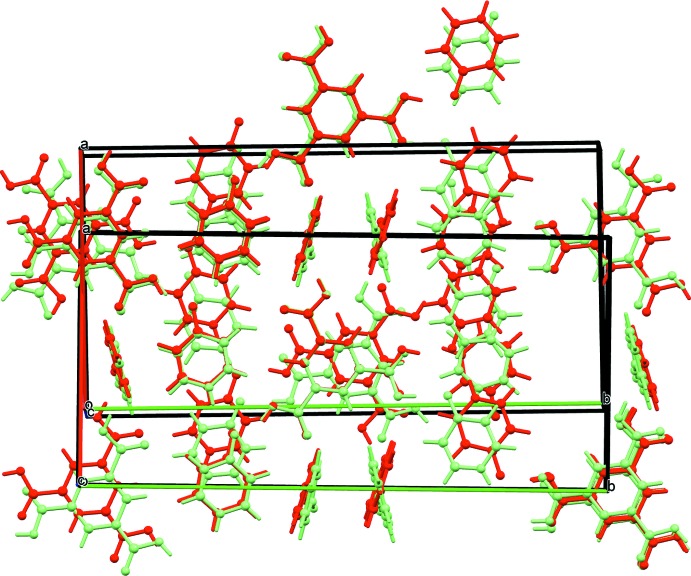
Overlay of the title compound (red) with the Campos-Gaxiola (light green) structure. The BTC moiety is used as the target for overlay. The view is along the *c* axis of both structures. Non-H atoms depicted as arbitrary spheres, H atoms as short sticks.

Inspection of an overlay of the two structures reveals some differences between the two polymorphs (Fig. 3 ▸). The orientation of the carb­oxy­lic acid groups of the BTC in the title compound has one ‘reversed’ with respect to the others, while the Campos-Gaxiola structure has all three oriented in the same direction, forming a propeller-like motif about the BTC. This results in a change in the hydrogen-bonding motif, reversing the orientations of the pyridone moieties. Perhaps the most prominent structural change is the orientation of the pyridone perpendicular to the plane of the BTC. In the title compound the pyridone rings are oriented with planes that are parallel to each other along the channels they occupy and are related by the screw axis parallel to the *c* axis. The perpendicular pyridone rings in the Campos-Gaxiola structure alternate their orientation along the channel, related by the *c*-glide. The change in hydrogen-bonding directionality is propagated to the orientation of the N1 and N2 pyridone chains. Examining the orientation of the carbonyl of the pyridone in these two chains reveals that the Campos-Gaxiola structure has the N1 and N2 chains oriented with the carbonyl along the *a*-axis forming a ‘parallel‘ alignment of the adjacent pyridone chains; again the *c*-glide is the cause for this arrangement. The N1 and N2 chains in the title compound adopt an ‘anti-parallel’ orientation with carbonyls in one chain being oriented in the opposite direction to the next chain, again a function of the screw axis. This is highlighted in Fig. 3 ▸ with the pyridone chain on the left of the figure showing an overlap of the pyridone rings between the two structures and the chain on the right of the figure showing the opposite orientation of the pyridone rings.

## Synthesis and crystallization   

The compound was formed by dissolving 4-hy­droxy­pyridine (0.112 g, 1.18 mmol) in methanol (3 mL) and benzene 1,3,5-tri­carb­oxy­lic acid (0.052 g, 0.24 mmol) in methanol (3 mL). The two solutions were combined and allowed to evaporate over 5 d yielding crystals suitable for diffraction studies. The crystallization process yields crystals of both the previously reported 1:1 co-crystal (Staun & Oliver, 2015 ▸) and those of the title compound. Presumably the differences in solvent composition and time for crystallization can yield one polymorph over the other. Several crystallization attempts were made using the methodology described herein (slow evaporation from methanol) and all yielded mixtures of the 1:1 and the 3:1 co-crystals reported herein. No evidence of the Campos-Gaxiola structure was observed within the crystals examined (reported as colorless rectangular prisms).

## Refinement   

Crystal data, data collection and structure refinement details are summarized in Table 3 ▸. Where possible, hydrogen atoms were initially located from a difference Fourier map and were subsequently refined using a riding model with C—H = 0.95 Å, N—H = 0.88 Å and O—H = 0.84 Å. *U*_iso_(H) was set to 1.2*U*_eq_(C/N) and 1.5*U*_eq_(O). The reliability for the correct enanti­omorph of the space group is low, due to the use of Mo *K*α radiation with a light atom structure. Analysis of the Flack *x* [0.1 (10); Flack, 1983 ▸], Hooft *y* [0.2 (10); Hooft *et al.*, 2008 ▸] and Parsons *z* [−0.2 (12); Parsons *et al.*, 2013 ▸] parameters tends to indicate that the correct enanti­omorph of the space group and absolute structure has been determined (Flack & Bernardinelli, 1999 ▸). Since these values are not close to zero the model could be refined as a racemic twin. However, this does not yield new or useful information and we have retained the standard model.

## Supplementary Material

Crystal structure: contains datablock(s) I, global. DOI: 10.1107/S2056989015017867/zl2644sup1.cif

Structure factors: contains datablock(s) I. DOI: 10.1107/S2056989015017867/zl2644Isup2.hkl

CCDC reference: 1427116

Additional supporting information:  crystallographic information; 3D view; checkCIF report

## Figures and Tables

**Table 1:** Pyridone / BTC interplanar angles ()

Pyridone ring	This work	Campos-Gaxiola
N1	7.3(2)	12.9
N2	8.5(2)	13.2
N3	87.5(3)	87.1

**Table 2:** Hydrogen-bond geometry (, )

*D*H*A*	*D*H	H*A*	*D* *A*	*D*H*A*
N1H1*N*O1^i^	0.88	1.89	2.762(8)	169
N2H2*N*O2^ii^	0.88	1.90	2.711(8)	152
N3H3*N*O3^iii^	0.88	2.01	2.773(10)	144
N3H3*N*O6^iv^	0.88	2.59	3.124(9)	120
O5H5*O*O1	0.84	1.75	2.555(7)	161
O7H7*O*O2	0.84	1.73	2.463(7)	145
O9H9*O*O3	0.84	1.70	2.526(7)	167
C1H1O4^i^	0.95	2.38	3.227(10)	148
C4H4O5	0.95	2.53	3.174(9)	126
C6H6O7^ii^	0.95	2.26	3.051(9)	140
C7H7O8^ii^	0.95	2.66	3.530(9)	153
C9H9O3^v^	0.95	2.58	3.227(9)	126
C11H11O6^iv^	0.95	2.46	3.076(11)	123
C11H11O9^vi^	0.95	2.55	3.159(9)	122
C12H12O6^vii^	0.95	2.49	3.302(11)	143
C14H13O4^viii^	0.95	2.60	3.405(10)	143
C15H15O8^iii^	0.95	2.66	3.608(10)	178

Symmetry codes: (i) 
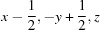
; (ii) 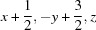; (iii) 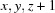; (iv) 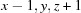; (v) 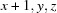; (vi) 
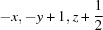
; (vii) 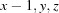; (viii) 
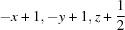
.

**Table 3:** Experimental details

Crystal data	
Chemical formula	C_9_H_6_O_6_3C_5_H_5_NO
*M*_r_	495.44
Crystal system, space group	Orthorhombic, *P* *n* *a*2_1_
Temperature (K)	120
*a*, *b*, *c* ()	12.699(3), 26.498(6), 6.6591(14)
*V* (^3^)	2240.9(8)
*Z*	4
Radiation type	Mo *K*
(mm^1^)	0.11
Crystal size (mm)	0.11 0.07 0.05

Data collection	
Diffractometer	Bruker APEXII
Absorption correction	Multi-scan (*SADABS*; Krause *et al.*, 2015 ▸)
*T*_min_, *T*_max_	0.647, 0.745
No. of measured, independent and observed [*I* > 2(*I*)] reflections	19034, 3257, 2418
*R*_int_	0.109
_max_ ()	23.4
(sin /)_max_ (^1^)	0.558

Refinement	
*R*[*F* ^2^ > 2(*F* ^2^)], *wR*(*F* ^2^), *S*	0.069, 0.171, 1.04
No. of reflections	3257
No. of parameters	328
No. of restraints	1
H-atom treatment	H-atom parameters constrained
_max_, _min_ (e ^3^)	0.43, 0.43

Computer programs: *APEX2* and *SAINT* (Bruker 2012 ▸), *SHELXS97* (Sheldrick, 2008 ▸), *SHELXL2014*/7 (Sheldrick, 2015 ▸), *OLEX2* (Dolomanov *et al.*, 2009 ▸), *Mercury* (Macrae *et al.*, 2008 ▸) and *publCIF* (Westrip, 2010 ▸).

## supplementary crystallographic information

### Crystal data

**Table:**

C_9_H_6_O_6_·3C_5_H_5_NO	*D*_x_ = 1.469 Mg m^−^^3^
*M**_r_* = 495.44	Mo *K*α radiation, λ = 0.71073 Å
Orthorhombic, *P**n**a*2_1_	Cell parameters from 1078 reflections
*a* = 12.699 (3) Å	θ = 3.1–19.2°
*b* = 26.498 (6) Å	µ = 0.11 mm^−^^1^
*c* = 6.6591 (14) Å	*T* = 120 K
*V* = 2240.9 (8) Å^3^	Rod, colorless
*Z* = 4	0.11 × 0.07 × 0.05 mm
*F*(000) = 1032	

### Data collection

**Table:**

Bruker APEXII diffractometer	3257 independent reflections
Radiation source: fine-focus sealed tube	2418 reflections with *I* > 2σ(*I*)
Graphite monochromator	*R*_int_ = 0.109
Detector resolution: 8.33 pixels mm^-1^	θ_max_ = 23.4°, θ_min_ = 1.5°
combination of ω and φ–scans	*h* = −14→14
Absorption correction: multi-scan (*SADABS*; Krause *et al.*, 2015)	*k* = −29→29
*T*_min_ = 0.647, *T*_max_ = 0.745	*l* = −7→7
19034 measured reflections	

### Refinement

**Table:**

Refinement on *F*^2^	Secondary atom site location: difference Fourier map
Least-squares matrix: full	Hydrogen site location: inferred from neighbouring sites
*R*[*F*^2^ > 2σ(*F*^2^)] = 0.069	H-atom parameters constrained
*wR*(*F*^2^) = 0.171	*w* = 1/[σ^2^(*F*_o_^2^) + (0.1002*P*)^2^] where *P* = (*F*_o_^2^ + 2*F*_c_^2^)/3
*S* = 1.04	(Δ/σ)_max_ < 0.001
3257 reflections	Δρ_max_ = 0.43 e Å^−^^3^
328 parameters	Δρ_min_ = −0.43 e Å^−^^3^
1 restraint	Absolute structure: Flack *x* determined using 801 quotients [(*I*^+^)-(*I*^-^)]/[(*I*^+^)+(*I*^-^)] (Parsons *et al.*, 2013).
Primary atom site location: structure-invariant direct methods	Absolute structure parameter: 0.1 (10)

### Special details

**Table:**

Geometry. All e.s.d.'s (except the e.s.d. in the dihedral angle between two l.s. planes) are estimated using the full covariance matrix. The cell e.s.d.'s are taken into account individually in the estimation of e.s.d.'s in distances, angles and torsion angles; correlations between e.s.d.'s in cell parameters are only used when they are defined by crystal symmetry. An approximate (isotropic) treatment of cell e.s.d.'s is used for estimating e.s.d.'s involving l.s. planes.

### Fractional atomic coordinates and isotropic or equivalent isotropic displacement parameters (Å^2^)

**Table:**

	*x*	*y*	*z*	*U*_iso_*/*U*_eq_	
O1	0.4424 (4)	0.29680 (18)	0.8670 (10)	0.0258 (14)	
N1	0.1387 (5)	0.2479 (3)	0.8807 (11)	0.0282 (18)	
H1N	0.0730	0.2374	0.8841	0.034*	
C1	0.2175 (6)	0.2147 (3)	0.9013 (15)	0.029 (2)	
H1	0.2017	0.1799	0.9202	0.035*	
C2	0.3205 (6)	0.2300 (3)	0.8955 (14)	0.028 (2)	
H2	0.3754	0.2059	0.9094	0.034*	
C3	0.3455 (5)	0.2814 (3)	0.8692 (14)	0.023 (2)	
C4	0.2598 (6)	0.3152 (3)	0.8499 (14)	0.029 (2)	
H4	0.2720	0.3503	0.8335	0.035*	
C5	0.1594 (6)	0.2968 (3)	0.8551 (15)	0.030 (2)	
H5	0.1023	0.3197	0.8400	0.036*	
O2	0.7873 (4)	0.71124 (18)	0.8498 (10)	0.0258 (14)	
N2	1.1005 (5)	0.7401 (2)	0.8856 (11)	0.0268 (18)	
H2N	1.1684	0.7465	0.8913	0.032*	
C6	1.0301 (6)	0.7782 (3)	0.9018 (15)	0.029 (2)	
H6	1.0550	0.8116	0.9216	0.034*	
C7	0.9253 (6)	0.7700 (3)	0.8907 (14)	0.025 (2)	
H7	0.8779	0.7976	0.9023	0.030*	
C8	0.8859 (5)	0.7208 (3)	0.8619 (14)	0.0216 (19)	
C9	0.9618 (5)	0.6815 (3)	0.8493 (14)	0.026 (2)	
H9	0.9392	0.6476	0.8330	0.031*	
C10	1.0663 (6)	0.6920 (3)	0.8605 (14)	0.028 (2)	
H10	1.1161	0.6654	0.8507	0.034*	
O3	0.0529 (4)	0.56842 (19)	0.8945 (9)	0.0217 (14)	
N3	−0.0040 (5)	0.5697 (3)	1.4925 (12)	0.0283 (19)	
H3N	−0.0161	0.5691	1.6226	0.034*	
C11	−0.0812 (6)	0.5564 (3)	1.3647 (15)	0.023 (2)	
H11	−0.1479	0.5468	1.4167	0.028*	
C12	−0.0659 (7)	0.5566 (3)	1.1636 (14)	0.025 (2)	
H12	−0.1218	0.5476	1.0756	0.030*	
C13	0.0335 (6)	0.5702 (3)	1.0850 (13)	0.020 (2)	
C14	0.1109 (6)	0.5847 (3)	1.2259 (13)	0.023 (2)	
H13	0.1780	0.5954	1.1794	0.027*	
C15	0.0916 (7)	0.5838 (3)	1.4248 (13)	0.022 (2)	
H15	0.1453	0.5931	1.5172	0.026*	
O4	0.6262 (4)	0.39539 (18)	0.7880 (11)	0.0327 (16)	
O5	0.4504 (3)	0.39158 (18)	0.7983 (10)	0.0270 (14)	
H5O	0.4625	0.3607	0.8143	0.040*	
O6	0.7915 (4)	0.57133 (19)	0.7531 (10)	0.0257 (14)	
O7	0.6792 (4)	0.63488 (19)	0.8051 (13)	0.046 (2)	
H7O	0.7346	0.6513	0.8273	0.069*	
O8	0.2964 (4)	0.61537 (19)	0.7789 (11)	0.0293 (14)	
O9	0.2359 (4)	0.53621 (19)	0.8195 (9)	0.0261 (15)	
H9O	0.1794	0.5512	0.8474	0.039*	
C16	0.5281 (5)	0.4719 (3)	0.7808 (14)	0.0161 (17)	
C17	0.6164 (5)	0.5025 (3)	0.7756 (13)	0.0196 (19)	
H17	0.6845	0.4878	0.7736	0.023*	
C18	0.6062 (5)	0.5544 (3)	0.7733 (13)	0.0163 (17)	
C19	0.5061 (6)	0.5761 (3)	0.7777 (14)	0.0200 (18)	
H19	0.4990	0.6118	0.7774	0.024*	
C20	0.4175 (5)	0.5460 (3)	0.7823 (13)	0.0177 (18)	
C21	0.4275 (6)	0.4933 (3)	0.7853 (14)	0.0195 (18)	
H21	0.3667	0.4725	0.7903	0.023*	
C22	0.5408 (6)	0.4164 (3)	0.7873 (14)	0.0229 (19)	
C23	0.3122 (5)	0.5701 (3)	0.7927 (13)	0.021 (2)	
C24	0.7028 (6)	0.5873 (3)	0.7757 (14)	0.0204 (18)	

### Atomic displacement parameters (Å^2^)

**Table:**

	*U*^11^	*U*^22^	*U*^33^	*U*^12^	*U*^13^	*U*^23^
O1	0.011 (3)	0.021 (3)	0.046 (4)	−0.002 (2)	0.004 (3)	0.002 (3)
N1	0.013 (3)	0.029 (4)	0.043 (5)	−0.010 (3)	0.004 (4)	−0.003 (4)
C1	0.024 (5)	0.025 (5)	0.040 (6)	−0.008 (4)	−0.001 (4)	−0.001 (5)
C2	0.024 (5)	0.024 (5)	0.037 (6)	0.002 (4)	0.005 (4)	0.001 (4)
C3	0.016 (4)	0.024 (4)	0.028 (5)	0.001 (4)	−0.001 (4)	−0.008 (4)
C4	0.021 (4)	0.026 (5)	0.039 (6)	−0.005 (4)	0.000 (5)	0.001 (5)
C5	0.021 (4)	0.030 (5)	0.038 (6)	0.002 (4)	−0.002 (4)	0.001 (5)
O2	0.013 (3)	0.022 (3)	0.042 (4)	0.000 (2)	−0.004 (3)	−0.003 (3)
N2	0.012 (3)	0.030 (4)	0.038 (5)	−0.003 (3)	−0.003 (4)	0.002 (4)
C6	0.018 (5)	0.021 (5)	0.046 (6)	−0.006 (4)	0.000 (4)	0.003 (5)
C7	0.023 (5)	0.014 (4)	0.037 (5)	0.001 (3)	0.001 (4)	0.006 (4)
C8	0.011 (4)	0.028 (5)	0.026 (5)	−0.005 (3)	−0.006 (4)	0.002 (4)
C9	0.020 (4)	0.014 (4)	0.044 (6)	−0.003 (3)	0.002 (4)	0.001 (4)
C10	0.021 (4)	0.025 (5)	0.040 (6)	0.000 (4)	0.004 (4)	0.001 (5)
O3	0.018 (3)	0.025 (3)	0.023 (4)	0.004 (2)	0.001 (3)	0.000 (3)
N3	0.042 (5)	0.019 (4)	0.024 (4)	0.003 (4)	0.001 (4)	−0.005 (3)
C11	0.012 (4)	0.022 (5)	0.036 (6)	0.000 (3)	0.003 (4)	0.001 (5)
C12	0.024 (5)	0.017 (5)	0.033 (6)	0.003 (4)	−0.003 (4)	−0.007 (4)
C13	0.024 (5)	0.006 (4)	0.028 (6)	0.005 (4)	0.001 (4)	0.000 (4)
C14	0.011 (4)	0.026 (5)	0.031 (6)	0.002 (4)	−0.002 (4)	0.000 (4)
C15	0.018 (5)	0.024 (5)	0.022 (5)	0.001 (4)	−0.007 (4)	0.001 (4)
O4	0.012 (3)	0.023 (3)	0.063 (5)	0.005 (2)	0.006 (3)	0.004 (4)
O5	0.012 (3)	0.018 (3)	0.050 (4)	−0.003 (2)	0.000 (3)	0.006 (4)
O6	0.009 (3)	0.025 (3)	0.043 (4)	0.000 (2)	0.001 (3)	−0.003 (3)
O7	0.013 (3)	0.021 (3)	0.106 (6)	−0.004 (2)	0.011 (4)	−0.011 (4)
O8	0.016 (3)	0.019 (3)	0.053 (4)	0.001 (2)	0.000 (3)	−0.004 (3)
O9	0.013 (3)	0.022 (3)	0.043 (4)	−0.001 (2)	0.006 (3)	0.003 (3)
C16	0.010 (4)	0.016 (4)	0.022 (4)	0.004 (3)	−0.003 (4)	−0.007 (4)
C17	0.010 (4)	0.027 (4)	0.021 (5)	0.003 (3)	0.000 (4)	−0.003 (4)
C18	0.011 (4)	0.015 (4)	0.022 (4)	0.002 (3)	0.002 (4)	−0.006 (4)
C19	0.016 (4)	0.019 (4)	0.025 (5)	0.000 (3)	0.000 (4)	0.003 (4)
C20	0.011 (4)	0.014 (4)	0.028 (5)	0.000 (3)	−0.003 (4)	−0.002 (4)
C21	0.013 (4)	0.022 (4)	0.024 (5)	−0.003 (3)	0.001 (4)	0.004 (4)
C22	0.021 (5)	0.023 (4)	0.025 (5)	0.000 (4)	0.003 (4)	−0.005 (5)
C23	0.011 (4)	0.021 (5)	0.030 (6)	−0.005 (3)	−0.003 (4)	0.000 (4)
C24	0.022 (5)	0.016 (4)	0.023 (5)	−0.001 (3)	0.002 (4)	0.002 (4)

### Geometric parameters (Å, º)

**Table:**

O1—C3	1.295 (8)	C11—H11	0.9500
N1—C5	1.334 (10)	C12—C13	1.413 (12)
N1—C1	1.340 (10)	C12—H12	0.9500
N1—H1N	0.8800	C13—C14	1.413 (12)
C1—C2	1.371 (11)	C14—C15	1.347 (12)
C1—H1	0.9500	C14—H13	0.9500
C2—C3	1.410 (11)	C15—H15	0.9500
C2—H2	0.9500	O4—C22	1.220 (8)
C3—C4	1.414 (10)	O5—C22	1.324 (8)
C4—C5	1.365 (10)	O5—H5O	0.8400
C4—H4	0.9500	O6—C24	1.213 (9)
C5—H5	0.9500	O7—C24	1.310 (9)
O2—C8	1.280 (8)	O7—H7O	0.8400
N2—C6	1.353 (10)	O8—C23	1.221 (8)
N2—C10	1.356 (10)	O9—C23	1.332 (8)
N2—H2N	0.8800	O9—H9O	0.8400
C6—C7	1.350 (11)	C16—C17	1.385 (9)
C6—H6	0.9500	C16—C21	1.398 (9)
C7—C8	1.410 (10)	C16—C22	1.481 (10)
C7—H7	0.9500	C17—C18	1.380 (9)
C8—C9	1.421 (10)	C17—H17	0.9500
C9—C10	1.358 (10)	C18—C19	1.395 (10)
C9—H9	0.9500	C18—C24	1.506 (10)
C10—H10	0.9500	C19—C20	1.380 (10)
O3—C13	1.293 (10)	C19—H19	0.9500
N3—C11	1.345 (10)	C20—C21	1.401 (10)
N3—C15	1.348 (10)	C20—C23	1.484 (10)
N3—H3N	0.8800	C21—H21	0.9500
C11—C12	1.353 (12)		
			
C5—N1—C1	120.3 (7)	C11—C12—C13	119.7 (8)
C5—N1—H1N	119.8	C11—C12—H12	120.2
C1—N1—H1N	119.8	C13—C12—H12	120.2
N1—C1—C2	121.0 (8)	O3—C13—C12	121.6 (8)
N1—C1—H1	119.5	O3—C13—C14	121.9 (8)
C2—C1—H1	119.5	C12—C13—C14	116.4 (8)
C1—C2—C3	120.4 (7)	C15—C14—C13	121.4 (9)
C1—C2—H2	119.8	C15—C14—H13	119.3
C3—C2—H2	119.8	C13—C14—H13	119.3
O1—C3—C2	121.3 (7)	C14—C15—N3	119.8 (8)
O1—C3—C4	122.0 (7)	C14—C15—H15	120.1
C2—C3—C4	116.7 (7)	N3—C15—H15	120.1
C5—C4—C3	119.4 (7)	C22—O5—H5O	109.5
C5—C4—H4	120.3	C24—O7—H7O	109.5
C3—C4—H4	120.3	C23—O9—H9O	109.5
N1—C5—C4	122.2 (7)	C17—C16—C21	120.2 (6)
N1—C5—H5	118.9	C17—C16—C22	119.7 (6)
C4—C5—H5	118.9	C21—C16—C22	120.1 (6)
C6—N2—C10	120.0 (6)	C18—C17—C16	120.5 (7)
C6—N2—H2N	120.0	C18—C17—H17	119.7
C10—N2—H2N	120.0	C16—C17—H17	119.7
C7—C6—N2	121.8 (8)	C17—C18—C19	119.7 (7)
C7—C6—H6	119.1	C17—C18—C24	120.0 (6)
N2—C6—H6	119.1	C19—C18—C24	120.2 (6)
C6—C7—C8	120.4 (7)	C20—C19—C18	120.3 (7)
C6—C7—H7	119.8	C20—C19—H19	119.8
C8—C7—H7	119.8	C18—C19—H19	119.8
O2—C8—C7	122.6 (7)	C19—C20—C21	120.2 (6)
O2—C8—C9	121.0 (7)	C19—C20—C23	119.2 (6)
C7—C8—C9	116.4 (7)	C21—C20—C23	120.6 (6)
C10—C9—C8	120.6 (7)	C16—C21—C20	119.1 (6)
C10—C9—H9	119.7	C16—C21—H21	120.4
C8—C9—H9	119.7	C20—C21—H21	120.4
N2—C10—C9	120.8 (7)	O4—C22—O5	123.0 (7)
N2—C10—H10	119.6	O4—C22—C16	123.4 (7)
C9—C10—H10	119.6	O5—C22—C16	113.6 (6)
C11—N3—C15	121.2 (8)	O8—C23—O9	123.6 (6)
C11—N3—H3N	119.4	O8—C23—C20	124.6 (6)
C15—N3—H3N	119.4	O9—C23—C20	111.8 (6)
N3—C11—C12	121.4 (8)	O6—C24—O7	124.5 (7)
N3—C11—H11	119.3	O6—C24—C18	123.6 (7)
C12—C11—H11	119.3	O7—C24—C18	111.9 (6)
			
C5—N1—C1—C2	−0.3 (15)	C21—C16—C17—C18	−0.5 (14)
N1—C1—C2—C3	0.5 (15)	C22—C16—C17—C18	−178.8 (8)
C1—C2—C3—O1	178.7 (9)	C16—C17—C18—C19	0.4 (13)
C1—C2—C3—C4	0.1 (14)	C16—C17—C18—C24	177.7 (8)
O1—C3—C4—C5	−179.4 (9)	C17—C18—C19—C20	−0.6 (14)
C2—C3—C4—C5	−0.8 (14)	C24—C18—C19—C20	−177.8 (8)
C1—N1—C5—C4	−0.4 (15)	C18—C19—C20—C21	0.8 (14)
C3—C4—C5—N1	1.0 (15)	C18—C19—C20—C23	178.3 (8)
C10—N2—C6—C7	−1.1 (14)	C17—C16—C21—C20	0.7 (13)
N2—C6—C7—C8	0.2 (15)	C22—C16—C21—C20	179.0 (9)
C6—C7—C8—O2	−179.8 (9)	C19—C20—C21—C16	−0.9 (13)
C6—C7—C8—C9	1.0 (14)	C23—C20—C21—C16	−178.3 (8)
O2—C8—C9—C10	179.4 (9)	C17—C16—C22—O4	−0.2 (15)
C7—C8—C9—C10	−1.4 (14)	C21—C16—C22—O4	−178.4 (9)
C6—N2—C10—C9	0.7 (14)	C17—C16—C22—O5	177.9 (7)
C8—C9—C10—N2	0.6 (15)	C21—C16—C22—O5	−0.3 (13)
C15—N3—C11—C12	0.2 (13)	C19—C20—C23—O8	7.1 (15)
N3—C11—C12—C13	1.0 (14)	C21—C20—C23—O8	−175.5 (9)
C11—C12—C13—O3	176.6 (8)	C19—C20—C23—O9	−172.7 (8)
C11—C12—C13—C14	−2.2 (13)	C21—C20—C23—O9	4.7 (12)
O3—C13—C14—C15	−176.3 (8)	C17—C18—C24—O6	10.1 (14)
C12—C13—C14—C15	2.4 (13)	C19—C18—C24—O6	−172.7 (9)
C13—C14—C15—N3	−1.3 (14)	C17—C18—C24—O7	−169.9 (8)
C11—N3—C15—C14	−0.1 (13)	C19—C18—C24—O7	7.3 (12)

### Hydrogen-bond geometry (Å, º)

**Table:**

*D*—H···*A*	*D*—H	H···*A*	*D*···*A*	*D*—H···*A*
N1—H1*N*···O1^i^	0.88	1.89	2.762 (8)	169
N2—H2*N*···O2^ii^	0.88	1.90	2.711 (8)	152
N3—H3*N*···O3^iii^	0.88	2.01	2.773 (10)	144
N3—H3*N*···O6^iv^	0.88	2.59	3.124 (9)	120
O5—H5*O*···O1	0.84	1.75	2.555 (7)	161
O7—H7*O*···O2	0.84	1.73	2.463 (7)	145
O9—H9*O*···O3	0.84	1.70	2.526 (7)	167
C1—H1···O4^i^	0.95	2.38	3.227 (10)	148
C4—H4···O5	0.95	2.53	3.174 (9)	126
C6—H6···O7^ii^	0.95	2.26	3.051 (9)	140
C7—H7···O8^ii^	0.95	2.66	3.530 (9)	153
C9—H9···O3^v^	0.95	2.58	3.227 (9)	126
C11—H11···O6^iv^	0.95	2.46	3.076 (11)	123
C11—H11···O9^vi^	0.95	2.55	3.159 (9)	122
C12—H12···O6^vii^	0.95	2.49	3.302 (11)	143
C14—H13···O4^viii^	0.95	2.60	3.405 (10)	143
C15—H15···O8^iii^	0.95	2.66	3.608 (10)	178

Symmetry codes: (i) *x*−1/2, −*y*+1/2, *z*; (ii) *x*+1/2, −*y*+3/2, *z*; (iii) *x*, *y*, *z*+1; (iv) *x*−1, *y*, *z*+1; (v) *x*+1, *y*, *z*; (vi) −*x*, −*y*+1, *z*+1/2; (vii) *x*−1, *y*, *z*; (viii) −*x*+1, −*y*+1, *z*+1/2.
